# A Novel PRRT2 Variant in Chinese Patients Suffering from Paroxysmal Kinesigenic Dyskinesia with Infantile Convulsion

**DOI:** 10.1155/2020/2097059

**Published:** 2020-05-18

**Authors:** Salem Baldi, Jin-Ling Zhu, Qing-Yun Hu, Ju-Li Wang, Jin-Bo Zhang, Shu-Hong Zhang

**Affiliations:** ^1^Department of Biology, School of Basic Medicine, Jiamusi University, Jiamusi City, Heilongjiang Province, 154007, China; ^2^Department of Anatomy, School of Basic Medicine, Jiamusi University, Jiamusi City, Heilongjiang Province, 154007, China; ^3^Department of Paediatrics, Central Hospital of Jiamusi, Jiamusi City Heilongjiang Province, 154002, China

## Abstract

PRRT2 mutations are the major causative agent of paroxysmal kinesigenic dyskinesia with infantile convulsion (PKD/IC). The study is aimed at screening PRRT2 gene mutations in patients who suffered from PKD/IC in Chinese population. Thirteen Chinese patients with PKD/IC were screened randomly for coding exons of the PRRT2 gene mutation along with 50 ethnically coordinated control people. Nine (2 unaffected) and 4 of the patients showed familial PKD/IC and apparently sporadic cases, respectively. We identified 5 different PRRT2 mutations in 10 individuals, including 8 familial and 2 apparently sporadic cases. However, no mutations were found in the 50 ethnically matched controls. Unknown (novel) NM_145239.2:c.686G>A and previously reported NM_145239.2:c.743G>C variants were identified in two familial and sporadic patients. All affected members of family A showed mutation NM_145239.2:c.650_670delinsCAATGGTGCCACCACTGGGTTA. The previously identified NM_145239.2:c.412 C>G and NM_145239.2:c.709G>A variants are seen in two individuals assessed in family B. Other than the previously identified variants, some of the patients with PRRT2-PKD/IC showed a new PRRT2 substitution variant. Thus, the spectrum of PRRT2 variants is expanded. The possible role and probability of PRRT2 variants involved in PKD/IC are highlighted.

## 1. Introduction

The paroxysmal dyskinesia comprises movement disorders characterized by decrease voluntary movements and the presence of involuntary movements. Three main types are present and categorized according to duration and recurrence of the attacks, namely, paroxysmal kinesigenic dyskinesia (PKD), paroxysmal nonkinesigenic dyskinesia, and paroxysmal exercise-induced dyskinesia, which are exertion-induced. Initially, PKD was the only known disorder characterized by unilateral or bilateral involuntary movement induced by sudden movement from rest, such as starting to walk or ascending from the sitting position. The attacks last for less than 1 min in duration with no pain or loss of consciousness. PKD, convulsions, familial infantile, with paroxysmal choreoathetosis (PKD/IC) and seizures, and benign familial infantile 2 (BFIS2) were recently linked to a similar district region 16p12-q12 and considered to be allelic diseases [1]. The PRRT2 gene provides instruction for the creation of the PRRT2 protein. The synaptic function is regulated by this protein by interacting with the synptosomal-associated protein (SNAP25) and different molecules of the *α*-amino-3-hydroxy-5-methyl-4isoxzazolepropionic acid (AMPA) complex receptors. The structural changes in PRRT2 gene sequence, such as base pair substitution, insertion, and deletion, can generate different types of mutations, including missense and nonsense mutations. The PRRT2 gene situated on the chromosome 16p11.2 with 4 exons encodes a membrane protein and reportedly causes several nervous system diseases [3]. PRRT2 protein occurrence is important in developing the sensory system, which includes the base of the brain and ganglia, throughout the embryonic developmental stages [4]. The PRRT2 has been depicted in various independent groups of Asian, African, and Caucasian families affected with PKD, PKD/IC, and BFIS2 [5–12]. Moreover, other epileptic phenotypes, including febrile seizure-related epilepsy [13–15] and hemiplegic migraine [16–19], have been implicated. We investigated whether the PRRT2 mutations are involved in PKD/IC and discover possible novel genetic mutations. Understanding PRRT2 mutation may reveal new treatment modalities, which may allow physicians to adopt new strategies for effective and timed treatment.

## 2. Material and Methods

### 2.1. Patients

**Figure 1:**
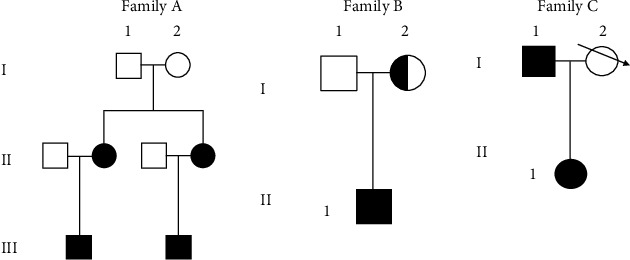
PKD/IC family pedigrees. The NM_1452392.2:c.650_670delinsCAATGGTGCCACCACTGGGTTA p.(Arg217Profs^∗^8) was found in family A. The NM_1452392.2:c.709G>A p.(Gly237Arg) was found in family B. The NM_1452392.2:c.686G>A p.(Arg229 Lys) was found in family C.

Thirteen individuals that comprise 9 familial cases (Figure 1) and 4 sporadic cases (Table 1) and 50 healthy controls were recruited from January 2016 to January 2017 in the ward of the Epilepsy Department of the Jiamusi Central Hospital. The controls were used to establish the baseline to compare the result of experiment's subjects and return valid results. All enrolled participants were personally interviewed and evaluated by at least two epileptologist. All the selected patients were new cases and met the diagnostic criteria of PKD set by Brun et al. These criteria include recognizing a kinesgenic trigger, having no pain or loss of consciousness during attacks that last less than 1 minute, and having favorable response to antiepileptic medicated treatment. A case was excluded if the patient did not take the test for PRRT2 gene and refused to provide informed consent for the study. All enrolled patients provided a written consent form in order to utilize their DNA. The study was approved by Medical Ethics Committee of Jiamusi University.

### 2.2. Mutation Analysis

The peripheral blood samples were collected from each subject, and a BioTeke DNA blood kit was used according to the instructions of the manufacture (BioTeke Corporation, China) to extract genomic DNA. The National Centre for Biotechnology Information (NCBI) software was used to design gene-specific primers. Firstly, DNA was amplified using a Biosystem polymerase chain reaction (PCR) over a number of cycles. PCR experiments were conducted with the following typical cycling conditions: initial denaturation at 95°C for 5 min, then 25 cycles of denaturation at 94°C for 30 s, annealing at 56°C for 30 s, extension at 72°C for 45 s, and final extension at 72°C for 5 min. We obtained the amplified PCR products that were separated on agarose gels and eventually sequenced using a DNA automated sequencer (Shenggong Bioengineering Co., Ltd., Shanghai, China) to confirm the presence of mutations. The methods were repeated three times and mutation confirmed by reverse sequence. Statistical analysis was performed using one-way ANOVA at the 5% level of significance. The obtained DNA sequences were contrasted with the genomic reference sequence of PRRT2 mRNA (NM_145239.2) published in NCBI. Thus, the disease-causing mutant sequence was determined. By the way, the reverse sequence was applied for the positive samples in order to confirm the presence of mutation. The description of variants and predicted proteins were described following current Human Genome Variation Society (HGVS) recommendations (http://www.hgvs.org/varnomen). The Mutalyzer was used to generate and check the HGVS variants description. (http://www.lovd.nl/mutalyzer). The well-described and reported variants were submitted to the PRRT2 gene variant database (Leiden Open Variation Database (LOVD)) [20–24].

### 2.3. Statistical Analysis

One-way analysis of variance (ANOVA), using Tukey test and NSTAT software was used to determine whether there are any statistically significant differences between the samples. Positive PKD patient group was compared with the negative group to determine the significance, at 0.05 significance level (Table 2).

The correlation and significance of collected data were evaluated by the IBM SPSS by one-way ANOVA. The significance was tested at 0.05 degrees of freedom, where simple *T* test was used to check the significance of the data.

## 3. Results

### 3.1. Clinical Features

The main manifestations of each family in our study are different from each other; the common clinical features of PKD, such as the duration of attacks, loss of consciousness during attacks, and good response to carbamazepine treatment, are shared in the three analyzed families. The significant difference between the group with and without PRRT2 gene mutations at the 0.05 level of significance (Table 2) was presented.

### 3.2. Genetic Finding

**Figure 2:**
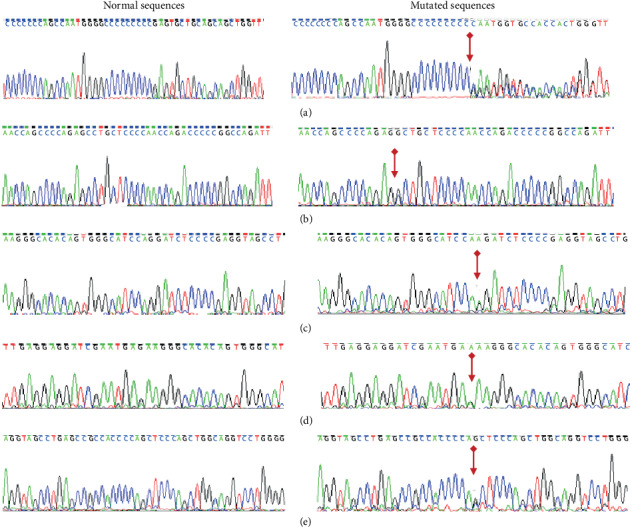
Five PRRT2 identified mutations in PKD/IC patients. (a) NM_1452392.2:c.650_670delinsCAATGGTGCCACCACTGGGTTA p.(Arg217Profs^∗^8); (b) NM_1452392.2:c.412C>G p.(Pro138Ala); (c) NM_1452392.2:c.709G>A p.(Gly237Arg); (d) NM_1452392.2:c.686G>A p.(Arg229Lys); and (e) NM_1452392.2:c.743G>C p.(Ser 248Thr).

The idiopathic PKD is a neurodevelopmental disorder firmly affected by genetic factors according to sequence analysis results. Table 1 provides a summary of the clinical features and detected PRRT2 mutations of all familial and sporadic patients suffering from PKD/IC. Eleven patients of PKD/IC, 2 unaffected relatives, and 50 healthy matched controls were analyzed for PRRT2 mutations. Several previously identified variants and one novel variant were obtained. Of the 3 families with PKD (7 patients, 2 unaffected), 8 individuals (88%) were identified with PRRT2 mutations. Out of the 4 sporadic cases, 2 (50%) demonstrated novel PRRT2 mutation. By using the direct sequence of the entire PRRT2 gene, one deletion-insertion mutation and 4 substitution mutations were identified (Figures 2(a)–2(e)) in 10 individuals, including 8 familial (one asymptomatic carrier) and 2 apparently sporadic cases (Table 1). NM_1452392.2: c.650_670delinsCAATGGTGCCACCACTGGGTTA p. (Arg217Profs^∗^8) mutation was identified in all patients from family A characterized by involuntary movements and responding appropriately to carbamazepine treatment (Figure 2(a)). The previously known variants of PRRT2 NM_1452392.2: c.412C>G p. (Prol138Ala) (Figure 2(b)) and PRRT2 NM_1452392.2: c.709G>A p. (Gly237Arg) (Figure 2(c)) were also identified in 2 individuals of family B. The previously unknown NM_1452392.2: c.686G>A p. (Arg229Lys) variant (Figure 2(d)) was detected in 6 individuals, of which 2 were in families B and C, and 2 sporadic cases. Polyphen-2 predicted the case to be benign. The PRRT2 NM_1452392.2: c.743G>C p. (Ser248Thr) (Figure 2(e)) was found in one patient of family C (Table 1). The severity prediction of NM_1452392.2: p. (Ser248Thr) is most likely harmed by the mutation taster polyphen-2. This finding reveals its pathogenic nature. None of the identified mutations were found in the 50 age-, sex-, and geographically matched healthy controls. Clinicogenetic associations analysis showed that there were no significant difference association of age of onset between patient who carried the mutation compared with those who did not *P* = 0.079 (Table 2). The presence of family history of PKD and history of involuntary movement was not significantly associated with the presence of PRRT2 mutation (Table 2).

## 4. Discussion

PKD is the most widely recognized type of paroxysmal dyskinesia characterized by involuntary convulsion and variability in manifestations [25]. PKD incidence is rare in the population, which accounted to 1/150,000 worldwide. Genome-wide linkage analysis and fine-mapping have enabled identification of susceptibility genes, including PRRT2 in PKD/IC appearance [26, 27]. The PRRT2 gene encodes transmembrane protein consisting of N-terminal extracellular and C-cytoplasmic domains [2]. Two recent studies have shown that the PRRT2 is profoundly expressed in neural cell development during the early embryonic stages. Thus, PRRT2 plays a major role in synaptic function. Reduced gene expression or changes in PRRT2 levels are correlated with alteration in neuronal function [28, 29]. Sanger sequencing was used to confirm the occurrence of PRRT2 mutation in 13 patients suffering from PKD/IC disorder in the Chinese cohort. The NM_1452392.2: c.650_670delinsCAATGGTGCCACCACTGGGTTA variant introducing a premature stop codon was found in all available members of family A. Thus, supreme cosegregation of PKD incident is shown within the family. This finding is consistent with the hereditary qualities, including the autosomal dominant property of PKD. The common NM_1452392.2: c.649dupC p. (Arg217Profs^∗^8) was previously identified in PKD/IC patients [4, 9, 30]. The NM_1452392.2:c.649delC and NM_1452392.2:c.649C>G mutations were previously identified in PKD European population [31].

The p.(Arg217Pfs^∗^8) accounts for 30.8% of sample population (44.4%, 4 out of 9 of our familial study population). Our finding and the finding of NM_1452392.2: c.649delC, NM_1452392.2: c.649C>G and NM_1452392.2: c.649dupC at the same position indicate the presence of a mutation hotspot in this region. This mutation may be due to slipped-strand mechanism during DNA replication. Thus, it is a hotspot mutation [8, 32]. The patient from family B (BII-1) presented a triple PRRT2 variants following genome sequencing of p.(Pro138Ala), p.(Arg229Lys), and p.(Gly237Arg) (Table 1). The double occurrence of PRRT2 mutations in a patient with PKD has been previously reported with intelligent disability [20]. While several studies reported that homozygous mutations cause sever neurological symptoms, recent reported c.981C>G homozygous mutation has not resulted in severe disorder [33]. Consistent with our result, this supports the hypothesis that differences in the epilepsy phenotypes in the patients with PRRT2 mutations depend on the types of PRRT2 mutations. We further screened parents of the patients for PRRT2 gene mutations and detected p.(Pro138Ala) and p.(Arg229Lys) variants in the unaffected mother of the patient (BI-2). This finding shows deficient mutation penetrance. However, the presence of p.(Gly237Arg) variant in both parents was not verified. The de novo result may endorse this mutation, which is consistent with genetic hereditary (Figure 1), in which putative mutation occurs de novo in the affected child of unaffected parents. Such a finding is in accordance with results of a previous study that presented the de novo mutagenesis of PRRT2 mutations in Chinese PKD, PKD/IC, and BFIS2 [32]. Moreover, p.(Pro138Ala) was reported in 15 Chinese sporadic PKD patients and in 14 patients affected with epileptic febrile seizures; its occurrence is seen as a loss of functional mutation [12]. PRRT2 p.(Pro138Ala) and p.(Gly237Arg) are predicted to be nonpathogenic and probably damaged by polyphen-2. Both are classified to be tolerated according to SIFT [2]. NM_1452392.2: c.412C>G is probably a causative mutation of PKD/IC. The proband in family C (CII-1) identified one previously unknown substitution variant NM_1452392.2: c.686G>A p.(Arg229 Lys) and previously reported NM_1452392.2: c.743G>C p.(Ser248Thr) variant (Table 1). The NM_1452392.2: c.686G>A p.(Arg229 Lys) is inherited from the father of the patient with cosegregation consistence. However, the NM_1452392.2: c.743G>C p.(Ser248Thr) variant was reported by the 1000 genome project, ExAC, HapMap, and Exome sequencing project (accession number rs 747687177). The unaffected mother passed away and was unavailable for sequence analysis. However, the pathogenicity of NM_1452392.2:c.686G>A p.(Arg229 Lys) cannot be proven and it is predicted to be benign by polyphen-2. Recent study reported a patient with a benign missense variant in the PRRT2 gene (c.501C>T; p.Thr167Ile). The variant was detected in the normal phenotype proband's father, but the proband showed infrequent clinical features that have not been previously reported in patients with the same mutation [34]. Consistently, our result promotes suggestion that an epigenetic role involvement during fetal development may result in severe clinical expressions [34]. The pathogenicity is more prevalent in PKD patients, and c.612dupA was found to cause loss of interaction between PRRT2 and syntaxin IB (STXIB) [35]. The cosegregation of NM_1452392.2:c.650_670delins CAATGGTGCCACCACTGGGTTA and NM_1452392.2:c.686G>A mutations in family A and family C is consistent with the autosomal dominant mode of inheritance of PKD/IC [2]. These results underpin the conceivable part of NM_1452392.2: c.686G>A in the pathogenicity. The PRRT2 variants may be involved in the aetiology of PKD/IC disease. Recent studies of PKD highlight the role of PRRT2 variants and possibly contribute to the pathogenesis of PKD [35, 36]. Additionally, there is a need to clarify the relation of p.(Arg229 Lys) with the pathogenesis of PKD/IC and with other PRRT2-related phenotypes. Only 3 individuals, including 1 unaffected familial and 2 sporadic patients, were negative for PRRT2 mutations in addition to 50 geographically healthy controls. Clearly, the sporadic negative screening patients were most likely misdiagnosed with PKD/IC because of clinical indications, or the patients possess a mutation in another gene [21]. The patients may possess a disease that is different from or similar to typical PKD/IC [6, 15, 32]. The study mainly researched on genomic mutations in PRRT2 gene and more causal mutations and new findings may be discovered if genomic variation from whole-exome sequencing data or whole-genome sequencing data is used in the future studies.

## 5. Conclusion

The study identified the genetic variations in PRRT2 associated with PKD/IC and uncovered new findings that indicate a novel mutation. Eleven patients of PKD/IC, i.e., 2 unaffected relatives and 50 healthy matched controls, were analyzed for PRRT2 mutations. Several previously identified variants and one novel variant were found.

In one family (A), the NM_1452392.2: c.650_670delins CAATGGTGCCACCACTGGGTTA PRRT2 mutation was identified as a causative mutation. Additionally, PRRT2 gene screen confirmed the presence of de novo mutation in the PKD patients, which may help clarify the mechanism of PKD. A novel sequence variant NM_1452392.2: c.686G>A p.(Arg229 Lys) in two families and two sporadic cases of PKD/IC are identified. These findings expand mutation spectrum of PRRT2. Detected PRRT2 mutations accounted for 88% and 50% in the investigated familial and sporadic cases of PKD/IC, respectively. Further studies involving functional experiments with more PKD pedigrees need to be conducted to reveal the pathogenicity of the detected variants and to clarify the molecular mechanisms by which these genetic variants alter human neural circuitry.

## Figures and Tables

**Table 1:** The clinical features and identified PRRT2 mutations of 13 sequenced individuals with PKD/IC.

Code number	Gender	Family/sporadic	Age at onset	Main symptoms	Drug treatment	PRTT2 mutation	Amino acids changes	Duration of attacks	Frequency of attacks	Involuntary movement
F1 (AIII-1)	M	Familial A	3 m	Upper limb dance-likemovement	CBZ	c.650_670delinsCAATGGTGCCACCACTGGGTTA	p.R217Pfs^∗^8	20 sec	10/d	>2 m
F2 (AIII-2)	M	Familial A	3 m	Upper limb dance-likemovement	CBZ	c.650_670delinsCAATGGTGCCACCACTGGGTTA	p.R217Pfs^∗^8	20 sec	10/d	>2 m
F3 (AII-1)	F	Familial A	20 y	Involuntary limb swinging	CBZ	c.650_670delinsCAATGGTGCCACCACTGGGTTA	p.R217Pfs^∗^8	20 sec	1/m	>20 y
F4 (AII-2)	F	Familial A	22 y	Involuntary limb swinging	CBZ	c.650_670delinsCAATGGTGCCACCACTGGGTTA	p.R217Pfs^∗^8	20 sec	1/m	>22 y
F5 (BI-2)	F	Familial B	No	No	No	c.412C>G c.686G>A	p. (Pro 138Ala) p. (Arg229 Lys)	No	No	No
F7 (BII-1)	M	Familial B	2 y	Upper limb movement	CBZ	c.412C>G c.686G>A c.709G>A	p. (Pro 138Ala) p. (Arg229 Lys) p. (Gly237Arg)	20 sec	5/d	>1 y
F13 (BI-1)	M	Familial B	No	No	No	Neg	Neg	No	No	No
F9 (CII-1)	F	Familial C	6 m	Sudden movement	CBZ	c.686G>A c.743G>C	p. (Arg229 Lys) p. (Ser248Thr)	18 sec	I/m	>4 m
F10 (CI-1)	M	Familial C	20	Sudden movement	CBZ	c.686G>A	p. (Arg229 Lys)	15 sec	1/m	>20 y
F8	F	Sporadic	2 y	Dystonia in lower limbs	CBZ	c.686G>A	p. (Arg229 Lys)	2 sec	4/d	>6 m
F11	M	Sporadic	11 y	Dystonia in lower limbs	CBZ	c.686G>A	p. (Arg229 Lys)	14 sec	5/d	>8 m
F16	F	Sporadic	6 y	Sudden movement	CBZ	Neg	Neg	15 sec	1/w	2 y
H1	F	Sporadic	4 y	Sudden movement	CBZ	Neg	Neg	55 sec	1/m	2 y

M: male; F: female; Y: year; No: normal; CBZ: carbamazepine; m: month; w: week; Neg: negative.

**Table 2:** Statistical analysis and clinical correlations.

Variable		Total (*N* = 13)	PRRT2 + VE(*N* = 10)	PRRT2-VE (*N* = 3)	Correlation	*P* value
Age of onset	Mean (SD)	8 y (1.318)	8.6 y (1.924)	5 y (2.775)	-0.3675	0.07958
	Median	6 y	2 y	5 y	—	—
Gender						
Male	*N* (%)	6 (46%)	5 (50%)	1 (33.3%)	-0.4158	0.2656
Female		7 (53.8%)	5 (50%)	2 (66.6%)	0.68047	0.3504
Origin (Chinese)	*N* (%)	13 (100%)	10 (77%)	3 (23%)	-0.3675	0.07958
Family history of PKD	*N* (%)	9 (69%)	8 (61.5%)	1 (7.7%)	-0.395563	0.0268
History of involuntary movement	*N* (%)	7 (53.8%)	5 (3.8%)	2 (15.3%)	-0.854242	0.0131
Duration of attack(s)	Mean Median	15 s 20 s	12.6 20 s	23.3 s 35 s	0.427347	0.0225
Frequency of attacks						
1-5 per day	*N* (%)	5 (38.4%)	5 (50%)	0 (0%)	—	—
1-5 per week		1 (07%)	0 (0%)	1 (33.3%)	—	—
1-5 per month		5 (38.4%)	4 (40%)	1 (33.3%)	-0.5222	0.12103

Statistical analysis indicates that the significant difference is between positive and negative PKD patients at 0.05 significant level and there is no correlation observed between different variant samples of positive and negative PKD patients.

## Data Availability

The data are shared with other partners, and available via contacting corresponding author, Jin-Lin Zhu (13845414150@139.com)
